# An individual treatment effect approach to predict response to mineralocorticoid receptor antagonists in patients with heart failure and reduced ejection fraction

**DOI:** 10.1002/ejhf.70047

**Published:** 2025-09-16

**Authors:** Masatake Kobayashi, Kevin Duarte, João Pedro Ferreira, Guillaume Baudry, Luca Monzo, John J.V. McMurray, Dirk J Van Veldhuisen, Bertram Pitt, Faiez Zannad, Nicolas Girerd

**Affiliations:** ^1^ Université de Lorraine, INSERM, Centre d'Investigations Cliniques 1433, CHRU de Nancy, Inserm 1116 and INI‐CRCT (Cardiovascular and Renal Clinical Trialists) F‐CRIN Network Nancy France; ^2^ Department of Cardiology Tokyo Medical University Tokyo Japan; ^3^ Cardiovascular Research and Development Center, Department of Surgery and Physiology Faculty of Medicine of the University of Porto Porto Portugal; ^4^ BHF Cardiovascular Research Centre, Institute of Cardiovascular and Medical Sciences, University of Glasgow Glasgow UK; ^5^ Department of Cardiology University of Groningen Groningen The Netherlands; ^6^ Department of Medicine University of Michigan School of Medicine Ann Arbor MI USA

**Keywords:** Heart failure with reduced ejection fraction, Mineralocorticoid receptor antagonist, Spironolactone, Eplerenone, Prognosis

## Abstract

**Aims:**

Mineralocorticoid receptor antagonists (MRAs) are often underused in patients with heart failure (HF) and reduced ejection fraction (HFrEF). Individual treatment effect (ITE) may assist physicians in making timely decisions about which patients are the best suited for personalized therapy. We aimed at developing and validating a model to estimate ITE of MRAs in patients with HFrEF.

**Methods and results:**

RALES and EMPHASIS‐HF trials were the derivation trials used to estimate ITE of MRAs versus placebo on cardiovascular death or HF hospitalization in HFrEF over a 2‐year period using counterfactual random forest method. ITE prediction models were built using linear regression and applied to the EPHESUS trial in patients with left ventricular systolic dysfunction and/or HF after myocardial infarction. In the RALES and EMPHASIS‐HF trials (*n* = 3887), age, body weight, blood pressure, heart rate, hypertension and diabetes prevalence, stroke history, left ventricular ejection fraction, renal function, and serum sodium and potassium concentrations were identified to determine ITE scores (adjusted *R* ^2^ = 0.25). As ITE scores increased, hazard ratio for treatment effect decreased from 0.82 (95% confidence interval [CI] 0.67–1.02) at ITE score 5 to 0.47 (95% CI 0.35–0.63) at ITE score 20 (*p* for interaction = 0.014). In the EPHESUS trial (*n* = 6472), a similar pattern was observed, with greater treatment effects in patients with higher ITE scores (*p* for interaction = 0.007).

**Conclusions:**

In HFrEF across various clinical settings, our simple ITE model predicted individual responses to MRA therapy. Although treatment effects may be attenuated at lower ITE scores, point estimates with wide CIs still generally favour benefit, suggesting that these patients still benefit.

## Introduction

Mineralocorticoid receptor antagonists (MRAs) are a fundamentally important therapy for patients with heart failure (HF) and reduced ejection fraction (HFrEF),^1^, ^2^ and selected patients with left ventricular dysfunction after myocardial infarction (MI).^3^ The prior suggestion of a benefit with the steroidal MRA spironolactone in patients with HF and preserved ejection fraction (HFpEF) has recently been confirmed with the new non‐steroidal MRA finerenone.^4^, ^5^, ^6^

However, registries have consistently shown underuse of MRA therapy in patients with HF.^7^, ^8^, ^9^, ^10^ This likely reflects concerns about renal function deterioration, hyperkalaemia and low blood pressure, although in clinical trials, patients who experienced these adverse effects had similar prognostic benefits from MRA therapy compared to those who did not.^11^, ^12^, ^13^ This reluctance could be mitigated if individualized prediction of the effect of an MRA was possible, enabling more confident and targeted treatment decisions.

One possible approach to predicting treatment effects is the individual treatment effect (ITE), which estimates the patient‐specific difference in outcome probability between a scenario where the patient receives treatment and one where they do not. A model capable of predicting ITE could assist clinicians in making timely, evidence‐based decisions, identifying patients most likely to benefit from treatment while minimizing unnecessary risks.^14^, ^15^

We hypothesized that the ITE approach can identify patients who benefit most from MRA therapy. In the present analysis, based on data from landmark clinical trials for MRA, we aimed to develop a scoring system based on routine clinical variables to predict the ITE of MRA on HF events from HFrEF trials, and then validating it in patients with left ventricular systolic dysfunction after MI.

## Methods

### Derivation trials in heart failure with reduced ejection fraction (RALES and EMPHASIS‐HF)

The Randomized Aldactone Evaluation Study (RALES) trial was the first of a series of clinical trials to explore MRA benefits on mortality in patients with HFrEF, wherein 1663 patients with severe HFrEF (New York Heart Association [NYHA] class III/IV, left ventricular ejection fraction [LVEF] <35%) were randomized to receive either spironolactone 25 mg/day or placebo. The design and main results of the RALES trial have previously been reported.^1^ RALES demonstrated a significant benefit of spironolactone versus placebo with 30% risk reduction for all‐cause mortality as the primary outcome.

In the Eplerenone in Mild Patients Hospitalization and Survival Study in Heart Failure (EMPHASIS‐HF) trial, 2737 patients with HF and an LVEF ≤30% (or, if >30% to 35%, a QRS duration of >130 ms on electrocardiography), and NYHA class II were randomly assigned to receive eplerenone (up to 50 mg daily) or placebo. The design, patient eligibility criteria, study procedure and main results of the EMPHASIS‐HF trial have been previously reported.^2^ The EMPHASIS‐HF trial demonstrated a significant benefit of eplerenone versus placebo with 37% risk reduction for cardiovascular (CV) death or HF hospitalization as the primary outcome.

### Validation trial in left ventricular systolic dysfunction after myocardial infarction (EPHESUS)

The Eplerenone Post‐Acute Myocardial Infarction Heart Failure Efficacy and Survival Study (EPHESUS) trial enrolled 6632 patients with LVEF ≤40% after acute MI who had signs and symptoms of HF or diabetes. All patients were entered into the study from 3 to 14 days after MI and were randomly assigned to receive either eplerenone 25 mg/day or placebo. The design and main results of the EPHESUS trial have previously been reported.^3^ EPHESUS trial demonstrated a significant benefit of eplerenone versus placebo with 15% risk reduction for all‐cause death as the primary outcome.

### Predicting individual treatment effect

To create a scoring system for ITE of MRA therapy, the following 15 clinical variables were selected based on their availability in trials and their potential association with MRA therapy and prognosis: age, sex, body weight, a prevalence of hypertension, diabetes, atrial fibrillation, chronic obstructive pulmonary disease, a history of MI and stroke, systolic blood pressure, heart rate, LVEF, estimated glomerular filtration rate (eGFR, Chronic Kidney Disease Epidemiology Collaboration formula^16^), and serum potassium and sodium concentrations.

In the first step, we used the counterfactual random forest method to estimate the ITE using data from the derivation cohort.^17^ Briefly, two random survival forests (RSF) with survival composite endpoint (CV death or HF hospitalization) as dependent variable and baseline clinical variables as explanatory variables were fitted separately in each treatment group, one RSF in patients under placebo and one RSF in patients under MRA. We predicted the risk of CV death or HF hospitalization at 2 years for each patient using both its natural forest as well as its counterfactual forest. Because separate RSF were fitted in placebo and MRA groups, covariate–outcome associations were allowed to differ between treatment arms. Note that out‐of‐bag predictions were used whenever possible to improve the stability of estimated values. ITE was then defined as the difference in risk of CV death and hospitalization for HF at 2 years under both treatments, conditional on the observed covariates. In the second step, we used linear regression models to predict ITE from baseline clinical variables without knowing the treatment group. To consider potential non‐linear relationships between continuous variables and treatment effect, all continuous variables were modelled using restricted cubic splines with three knots (i.e. with one linear component and one cubic component) fixed at the 10th, 50th and 90th percentiles, according to Harrell's recommendation.^18^ Backward selection using Bayesian information criterion was performed to avoid overfitting and obtain a parsimonious model. Model performance was evaluated by calculating adjusted *R* ^2^.

### Statistical analysis

Baseline characteristics of the cohorts a described as mean ± standard deviation for continuous variables and as frequency (percentage) for categorical variables. Patients with missing values were excluded from the present analysis.

In the derivation cohort, we developed clinical scores to predict ITE of MRA therapy from baseline clinical variables using the methods described above. The association between ITE prediction and each continuous variable retained in the linear model was plotted for a hypothetical population where all other variables were set at the mean values observed in the derivation cohort.

Individual treatment effect was then predicted in each cohort using this clinical score. To prevent extrapolation beyond the range of values used in developing the model, continuous variables were winsorized. Specifically, the minimum and maximum values from the derivation cohort were used as thresholds—any values below the minimum or above the maximum were capped at these limits before applying the linear model to new individuals.^19^

Mineralocorticoid receptor antagonist treatment effect on composite endpoint was assessed in each cohort using Cox models with ITE prediction, treatment and interaction between the two terms as covariates. The proportional hazards assumption was verified in all models using Schoenfeld residuals, with no relevant violations detected. The effect of MRA according to the predicted value for ITE was plotted.

All statistical analyses were performed using the R software (The R Foundation for Statistical Computing, Vienna, Austria) version 4.1.1. RSF was performed using the function rfsrc in the R package RandomForestSRC. A *p*‐value <0.05 was considered statistically significant.

## Results

### Patient characteristics

Amongst the RALES, EMPHASIS‐HF and EPHESUS trials, patients included in RALES were the least likely to have prevalent hypertension, diabetes, and a history of MI, and had the lowest baseline eGFR levels and highest rate of CV death or HF hospitalization. Those included in EMPHASIS‐HF had highest frequency of atrial fibrillation (*Table* *1*).

**Table 1:** Baseline characteristics of the trials (RALES, EMPHASIS‐HF and EPHESUS)

	Derivation trials			Validation trial EPHESUS (*n* = 6472)
	Overall (*n* = 3887)	RALES (*n* = 1608)	EMPHASIS‐HF *n* = 2279)	
MRA allocation, *n* (%)	1935 (49.8)	791 (49.2)	1144 (50.2)	3243 (50.1)
Age, years	67.2 ± 9.8	65.2 ± 11.9	68.6 ± 7.6	63.9 ± 11.5
Male sex, *n* (%)	2936 (75.5)	1171 (72.8)	1765 (77.4)	4598 (71.0)
Weight, kg	75.8 ± 16.5	71.0 ± 14.9	79.2 ± 16.8	78.1 ± 15.1
Body mass index, kg/m^2^	27.5 ± 4.8	–	27.5 ± 4.8	27.4 ± 4.5
Smoking status, *n* (%)				
Never smoker	1031 (45.2)	–	1031 (45.2)	2523 (39.0)
Past smoker	1003 (44.0)	–	1003 (44.0)	2004 (31.0)
Current smoker	245 (10.8)	–	245 (10.8)	1945 (30.1)
Medical history, *n* (%)				
Hypertension	1917 (49.3)	379 (23.6)	1538 (67.5)	3906 (60.4)
Diabetes	1078 (27.7)	355 (22.1)	723 (31.7)	2078 (32.1)
Atrial fibrillation	883 (22.7)	166 (10.3)	717 (31.5)	849 (13.1)
Angina	1152 (29.6)	107 (6.7)	1045 (45.9)	2672 (41.3)
PCI and/or CABG	891 (22.9)	152 (9.5)	739 (32.4)	2934 (45.3)
Myocardial infarction	1643 (42.3)	461 (28.7)	1182 (51.9)	6472 (100.0)
Stroke	363 (9.3)	142 (8.8)	221 (9.7)	569 (8.8)
COPD	602 (15.5)	283 (17.6)	319 (14.0)	604 (9.3)
Anaemia	578 (25.4)	–	578 (25.4)	2133 (33.0)
Systolic blood pressure, mmHg	123.5 ± 18.3	122.2 ± 20.1	124.4 ± 16.8	119.1 ± 16.4
Heart rate, bpm	75.5 ± 13.8	80.9 ± 14.2	71.7 ± 12.2	74.7 ± 11.7
LVEF, %	25.8 ± 5.6	25.4 ± 6.7	26.1 ± 4.7	33.1 ± 6.0
eGFR, ml/min/1.73 m^2^	67.6 ± 22.0	62.6 ± 21.4	71.2 ± 21.7	68.4 ± 20.9
Potassium, mmol/L	4.3 ± 0.4	4.2 ± 0.4	4.3 ± 0.4	4.3 ± 0.4
Sodium, mmol/L	139.6 ± 4.0	138.7 ± 3.9	140.1 ± 4.0	139.5 ± 4.4
CV death, *n* (%)	836 (21.5)	564 (35.1)	272 (11.9)	850 (13.1)
HF hospitalization, *n* (%)	833 (21.4)	501 (31.2)	332 (14.6)	824 (12.7)
CV death/HF hospitalization, *n* (%)	1317 (33.9)	826 (51.4)	491 (21.5)	1392 (21.5)

Values are mean ± standard deviation for continuous variables and *n* (%) for categorical variables.

CABG, coronary artery bypass graft; COPD, chronic obstructive pulmonary disease; CV, cardiovascular; eGFR, estimated glomerular filtration rate; HF, heart failure; LVEF, left ventricular ejection fraction; MRA, mineralocorticoid receptor antagonist; PCI, percutaneous coronary intervention.

### Baseline characteristics for predicting individual treatment effect

**Figure 1:**
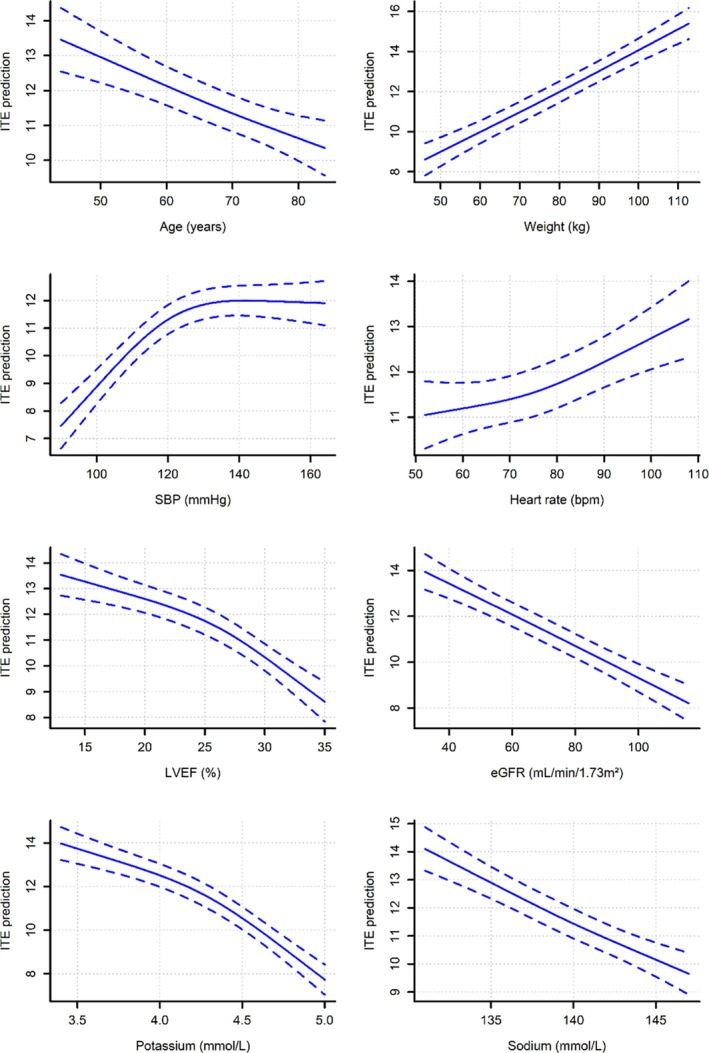
Relationship between predicted individual treatment effect (ITE) and continuous variables retained in the linear model. Solid lines represent the predictions from linear model for a hypothetical population where all other variables were set at the mean values observed in the derivation cohort. Dotted lines represent the 95% confidence interval around the regression curve. eGFR, estimated glomerular filtration rate; LVEF, left ventricular ejection fraction; SBP, systolic blood pressure.

In the RALES and EMPHASIS‐HF trials, we identified that 11 patient characteristics, including age, body weight, systolic blood pressure, heart rate, prevalent hypertension and diabetes, history of stroke, LVEF, eGFR, and sodium and potassium concentration were significantly associated with the effect of MRA therapy on the outcome (adjusted *R* ^2^ = 0.249) (*Table* *2*). Patients with diabetes had more than two‐fold higher ITE scores compared to those without diabetes. A stroke history was associated with higher ITE model score, while hypertension prevalence was associated with lower scores (*Table* *2*). Younger age, greater weight, higher heart rate, lower LVEF, eGFR, serum potassium and sodium concentrations were associated with higher ITE scores (*Figure* *1*). Higher systolic blood pressure was associated with higher ITE scores up to approximately 130 mmHg, beyond which the curve plateaued (*Figure* *1*).

**Table 2:** Linear regression model for individual treatment effect prediction

	Beta (SE)	*p*‐value
Intercept	51.627 (6.882)	<0.0001
Age (years)		
Linear	−0.083 (0.020)	<0.0001
Cubic	0.008 (0.025)	0.75
Weight (kg)		
Linear	0.098 (0.015)	<0.0001
Cubic	0.005 (0.016)	0.78
Systolic blood pressure (mmHg)		
Linear	0.142 (0.014)	<0.0001
Cubic	−0.117 (0.018)	<0.0001
Heart rate (bpm)		
Linear	0.018 (0.018)	0.33
Cubic	0.028 (0.024)	0.23
LVEF (%)		
Linear	−0.133 (0.037)	0.0004
Cubic	−0.111 (0.041)	0.007
eGFR (ml/min/1.73 m^2^)		
Linear	−0.067 (0.012)	<0.0001
Cubic	−0.002 (0.015)	0.90
Potassium (mmol/L)		
Linear	−2.356 (0.488)	<0.0001
Cubic	−2.140 (0.581)	0.0002
Sodium (mmol/L)		
Linear	−0.301 (0.047)	<0.0001
Cubic	0.030 (0.053)	0.57
Diabetes	2.403 (0.234)	<0.0001
Stroke	1.416 (0.352)	<0.0001
Hypertension	−0.953 (0.224)	<0.0001

Adjusted *R* ^2^ = 0.249 (adjusted *R* = 0.499).

eGFR, estimated glomerular filtration rate; LVEF, left ventricular ejection fraction; SE, standard error.

### Association of individual treatment effect predicted value with outcomes in placebo and mineralocorticoid receptor antagonist arms

In placebo arms, ITE was strongly associated with the composite outcome (hazard ratio [HR] per 5‐point increase: 1.40 [1.27–1.54] in RALES/EMPHASIS‐HF and 1.51 [1.38–1.65] in EPHESUS; both *p* < 0.0001), whereas the association was weaker in the MRA groups (HR 1.16 [1.03–1.30] and 1.26 [1.15–1.39], respectively) (online supplementary *Table* *S1* and *Figure* *S1*).

### Mineralocorticoid receptor antagonist efficacy according to individual treatment effect predicted value

**Figure 2:**
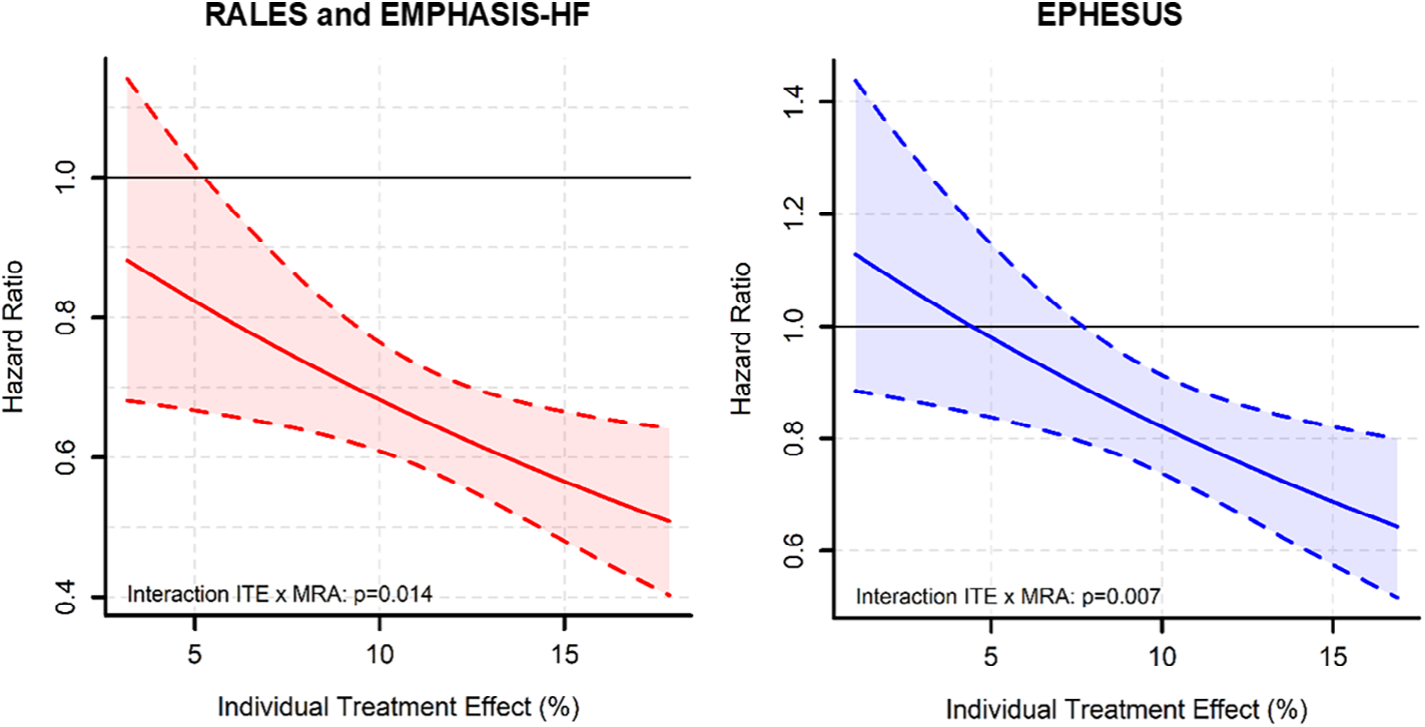
Effect of mineralocorticoid receptor antagonists (MRA) according to predicted individual treatment effect (ITE) value in the derivation cohorts (Randomized Aldactone Evaluation Study [RALES] and Eplerenone in Mild Patients Hospitalization and Survival Study in Heart Failure [EMPHASIS‐HF]) and in the validation cohort (Eplerenone Post‐Acute Myocardial Infarction Heart Failure Efficacy and Survival Study [EPHESUS]). Solid lines indicate the hazard ratio for the effect of MRA, and shaded polygons represent 95% confidence interval surrounding the hazard ratio.

In the RALES and EMPHASIS‐HF trials, patients with higher calculated ITE were more likely to experience a reduced risk of HF events from MRA (HR ranging from 1.06 to 0.40, *p* for interaction between ITE and MRA = 0.014) (*Figure* *2*). Patients with an ITE score of 5 had a HR of 0.82 (95% confidence interval [CI] 0.67–1.02), those with a score of 10 had an HR of 0.68 (95% CI 0.61–0.76), and those with a score of 15 had an HR of 0.57 (95% CI 0.48–0.67).

Correspondingly, in the EPHESUS trial, patients with higher ITE were more likely to benefit from MRA versus placebo (*p* for interaction between ITE and MRA = 0.007) (*Figure* *2*).

Importantly, the interaction between ITE and MRA remained consistently significant across cohorts, even when all ITE component variables were entered into the model. In contrast, interactions between individual components of the ITE and MRA were only sporadically significant (e.g. weight and systolic blood pressure in RALES/EMPHASIS‐HF, age in EPHESUS) and never reproduced across both derivation and validation cohorts (online supplementary *Table* *S2*).

In addition to relative treatment effects, we examined absolute risk reductions across ITE tertiles (online supplementary *Table* *S3*). In the derivation cohort (RALES/EMPHASIS‐HF), the absolute 2‐year risk reduction with MRA therapy ranged from −8.3% (95% CI –13.9 to −2.6) in the lowest tertile to −17.4% (95% CI –23.2 to −11.6) in the highest tertile. In the validation cohort (EPHESUS), the corresponding values ranged from −1.1% (95% CI –5.2 to 3.0) in the lowest tertile to −6.4% (95% CI –11.5 to −1.3) in the highest tertile (online supplementary *Table* *S3*).

### Examples of individual treatment effect prediction model

**Figure 3:**
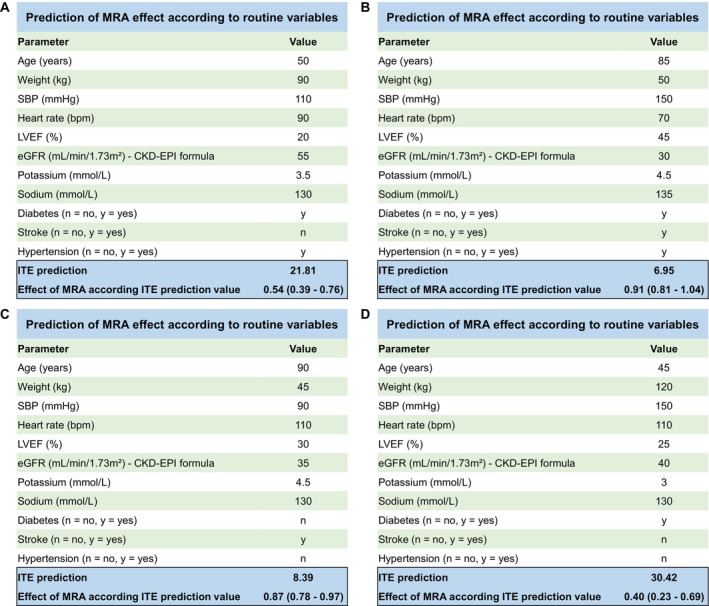
Examples of individual treatment effect (ITE) prediction models. CKD‐EPI, Chronic Kidney Disease Epidemiology Collaboration; eGFR, estimated glomerular filtration rate; LVEF, left ventricular ejection fraction; MRA; mineralocorticoid receptor antagonist; SBP, systolic blood pressure.

Examples of ITE prediction model (providing the estimations from the validation analysis in the EPHSESUS trial) are presented in *Figure* *3*. For example, a 50‐year‐old man with diabetes and hypertension, weighing 90 kg, and systolic blood pressure of 110 mmHg, heart rate of 90 bpm, LVEF of 20%, eGFR of 55 ml/min/1.73 m^2^, serum potassium of 3.5 mmol/L, and serum sodium of 130 mmol/L has a calculated ITE score of 21.8, corresponding to an estimated 46% reduction in HF‐related risk with MRA therapy (95% CI 24–61%).

A 85‐year‐old woman with a prevalence of diabetes and hypertension and a stroke history presented with a body weight of 50 kg, systolic blood pressure of 150 mmHg, heart rate of 70 bpm, LVEF of 45%, eGFR of 30 ml/min/1.73 m^2^, serum potassium of 4.5 mmol/L, and serum sodium of 135 mmol/L has a calculated ITE score of 7.0, corresponding to an estimated 9% reduction in HF‐related risk with MRA therapy (95% CI −4% to 19%).

A tool to calculate the ITE score for MRA therapy is available online (https://cic‐p‐nancy.fr/mra‐ite‐prediction‐calculator‐in‐hfref/).

## Discussion

Using data from the landmark randomized clinical trials of MRA therapy in HF, we developed a simple, clinician‐friendly model based on 11 key clinical features (e.g. body weight, systolic blood pressure, diabetes prevalence, renal function, and potassium concentrations). This model accurately predicted the effect of MRA therapy on HF outcomes among patients with HFrEF across diverse clinical settings (i.e. mild symptoms and severe symptoms, and post‐MI). By providing a practical decision‐support tool, this score has the potential to provide personalized decisions for MRA therapy in clinical practice. Beyond its potential clinical utility, this study serves as a compelling demonstration of the ITE model, showcasing its ability to predict trial outcomes in different settings.

In the present study, higher blood pressure and lower potassium were associated with higher ITE scores. Classically, aldosterone activation increases sodium reabsorption and blood pressure, while promoting reducing serum potassium via activation of the apical epithelial sodium channel (ENaC) and the basolateral Na/K‐ATPase in the distal nephron.^20^ In a sub‐analysis of the RALES and EMPHASIS‐HF trials, a risk reduction of HF‐related events with MRA therapy versus placebo was numerically greater in patients with higher blood pressure (>135 mmHg).^13^ In a sub‐analysis of the EMPHASIS‐HF trial, eplerenone mitigated HF‐related risk associated with baseline hypokalaemia compared to placebo (*p* for interaction = 0.04).

Greater body weight, likely reflecting adiposity, and diabetes were also associated with higher ITE scores. Obese patients may experience increased aldosterone secretion, triggered by renin–angiotensin–aldosterone system activation and leptin,^21^, ^22^ while mineralocorticoid receptor (MR) can also be activated by cortisol due to 11β‐HSD2 down‐regulation or ligand‐independent cytokines.^23^, ^24^ In a sub‐analysis of the RALES and EMPHASIS‐HF trials, risk reduction of HF‐related events with MRA therapy was larger with higher body weight regardless of sex difference.^25^ Furthermore, hyperglycemia induces protein kinase C signaling, leading to aldosterone activation and increased MR expression,^26^, ^27^ while MR activation impairs insulin sensitivity, leading to diabetes progression.^28^ A greater benefit from MRA therapy was observed in patients with diabetes, particularly those treated with insulin, in a sub‐analysis of the EMPHASIS‐HF trial.^29^

In our integrated ITE model, lower eGFR and younger age were associated with higher ITE scores. The association between kidney injury/dysfunction and MR overactivation has been well‐established,^20^, ^30^, ^31^ while patients with poor renal function may be at risk of severe congestion status and further aldosterone activation.^32^, ^33^, ^34^ In a meta‐analysis of the RALES, EMPHASIS‐HF, EPHESUS and TOPCAT Americas trials, benefits from MRA therapy was attenuated as eGFR decrease,^35^ which may reflect higher rates of MRA discontinuation and hyperkalaemia in patients with lower eGFR. However, these findings were mainly driven by patients with eGFR <30 ml/min/1.73 m^2^ (<3% of patients), while those with eGFR 30 to 60 ml/min/1.73 m^2^ still had ~20% risk reduction. Therefore, our ITE model may identify patients with lower eGFR (irrespective of comorbidities associated with eGFR) who are more likely to have advanced HF status and derive greater benefit from MRA therapy. Furthermore, the association between younger age and higher ITE score may be partly explained by the fact that age‐related physiological changes, comorbidities, frailty, polypharmacy, and competing risks may attenuate the potential efficacy of MRA therapy in older patients.^36^

Lower LVEF, which is associated with greater activation of the renin–angiotensin–aldosterone system in HFrEF,^32^ predicted higher ITE scores. Furthermore, higher heart rate and lower sodium concentration may reflect more advanced HF status, which potentially increases a greater benefit from MRA therapy.^37^, ^38^

To some extent, it was surprising that the scoring system derived from the RALES and EMPHASIS‐HF trials, which included patients with a broad severity of HFrEF, was successfully validated in patients with left ventricular systolic dysfunction after MI (in the EPHESUS trial). Reflecting the stages and severity of HF and background therapies, the mortality risk of patients in the placebo group varied substantially depending on the trials (the event rates per 100 person‐years = 26.6 [24.1–29.4] in RALES; 8.9 [7.8–10.2] in EMPHASIS‐HF; 13.0 [11.9–14.1] in EPHESUS).^1^, ^2^, ^3^ Despite these major differences, the predictive validity of our ITE model remained robust in patients with HFrEF across the diverse clinical contexts studied, including mild as well as severe symptoms and after acute MI.

In addition to relative treatment effects, our analyses also demonstrated clinically meaningful differences in absolute risk reduction across ITE strata. In both derivation and validation cohorts, patients with higher ITE scores experienced the largest absolute treatment gains, whereas those with low ITE scores derived little or no measurable absolute benefit (online supplementary *Table* *S3*). These findings confirm that patients with higher ITE scores not only exhibit stronger relative benefit, but also experience substantially greater absolute treatment effects. Since absolute benefit is the key driver of clinical decision‐making, the ITE model may serve as a companion tool to reassure prescribers that most patients are likely to derive meaningful benefit from MRA therapy, potentially helping to overcome therapeutic inertia and underuse.

Our ITE approach differs substantially from prior risk‐based subgroup analyses, such as the EMPHASIS‐HF substudy, which reported consistent relative benefit of MRA therapy across baseline risk groups.^39^ Rather than stratifying patients by baseline prognosis, ITE directly contrasts predicted outcomes under MRA and placebo at the individual level. This approach enables us to move beyond simple risk stratification and to investigate potential heterogeneity in relative treatment effects, rather than presuming uniform benefit across predefined risk categories.

Even individuals with low predicted treatment effect scores appeared to derive benefit from MRAs in this HFrEF population. This underscores that MRAs should remain broadly indicated and that underuse cannot be justified by an assumption of lack of efficacy in certain patients. Nevertheless, MRAs remain substantially underutilized in clinical practice,^7^, ^8^, ^9^, ^10^ a phenomenon that suggests lingering doubts among clinicians about their net benefit in some individuals. By providing patient‐level evidence that nearly all patients stand to gain, our findings may help counteract this skepticism and support wider implementation of MRA therapy.

Our analyses highlight that ITE conveys information beyond baseline risk stratification and beyond what can be captured by single covariates. The consistent significance of the ITE × MRA interaction across derivation and validation cohorts, coupled with the lack of reproducible interactions for individual components, suggests that ITE captures a higher‐order signal of treatment heterogeneity. This finding reinforces the potential of ITE as a more robust and clinically meaningful approach than conventional interaction analyses and supports its added value for personalizing therapy in HF.

A key advantage of our prediction model is its broad applicability, as it relies only on routinely available clinical information. This simplicity makes integration into electronic health records or mobile applications readily feasible.^40^, ^41^ It is already accessible online (https://cic‐p‐nancy.fr/mra‐ite‐prediction‐calculator‐in‐hfref/). Beyond this practical implementation, the study also illustrates the proof‐of‐concept value of individualized treatment effect prediction, highlighting how such approaches can reassure clinicians that nearly all patients stand to gain and thereby help overcome therapeutic inertia. More generally, ITE‐based profiling may be useful when trials show uncertain treatment response or potential subgroup heterogeneity, or when costly drugs with substantial side effects require careful patient selection. Our findings, therefore, may pave the way for a more personalized approach to MRA therapy in HF and enrich the design of more targeted future clinical trials.

### Limitations

The main limitations of this analysis were its post‐hoc nature and should be regarded as hypothesis‐generating.

The pivotal HFrEF trials informing this analysis were conducted more than two decades ago in populations not exposed to contemporary therapies such as sacubitril/valsartan or sodium–glucose co‐transporter 2 inhibitors, although they may not impact the effect of MRA therapy on HF events.^42^, ^43^ These landmark studies, however, remain the only randomized evidence base for MRAs in HFrEF and the foundation of current guideline recommendations. Our analysis should therefore be considered as leveraging this unique evidence to generate insights that help translate historical trial populations into the context of present‐day clinical practice. Importantly, the score system derived from the RALES and EMPHASIS‐HF trials was externally validated in other MRA trials for left ventricular systolic dysfunction, suggesting the possibility of implementing our score system into general populations with HF regardless of background therapies.

Both the derivation and validation cohorts had relatively short follow‐up durations. Although such horizons may favour variables with stronger short‐term prognostic influence and could affect the stability of ITE estimates, the consistent replication across cohorts supports the robustness of our findings.

Some of the CIs for the association between ITE and outcomes were wide, reflecting statistical uncertainty. This may limit precision at the individual level and underscores the need for further refinement and external validation before clinical translation. Nonetheless, the consistent ITE × MRA interaction across cohorts suggests that the overall findings are robust.

We selected body weight rather than body mass index, which was not available in the RALES dataset. Prior analyses and our own interaction models confirmed an association between body weight and MRA efficacy. However, the effect of body weight did not remain significant once ITE was considered, indicating that ITE captures the main relevant signals affecting heterogeneity of treatment effect. Body weight may thus act as a surrogate for multiple underlying factors, including sex and body size.

We did not considered HFpEF trials such as TOPCAT Americas or FINEARTS‐HF, as our aim was to focus exclusively on HFrEF, thus avoiding unmanageable heterogeneity.^41^, ^42^

Given the well‐established clinical benefits of MRA therapy in treating a broad range of cardiovascular and kidney diseases (e.g. HF and kidney disease),^44^, ^45^ whether our ITE model can also predict the efficacy of MRA therapy in these settings may enhance the robustness of our ITE approaches and merits further investigation.

## Conclusions

In HFrEF across various clinical settings (i.e. mild and severe symptoms, and post‐MI), we developed and validated a simple ITE model to predict individual responses to MRA therapy. Although treatment effects may be attenuated at lower ITE scores, point estimates—with wide CIs—still generally favour benefit, suggesting that most patients still benefit even at low ITE score. Notably, the variables predicting treatment effect, including greater body weight, poor kidney function, lower LVEF and diabetes, are all known to be associated with increased MR activations, thereby providing a strong mechanistic basis to support the clinical relevance of the model.

**Conflict of interest**: none declared.

## Supporting information


**Appendix S1.** Supporting Information.
