# Supplementary material for: An individual treatment effect approach to predict response to mineralocorticoid receptor antagonists in patients with heart failure and reduced ejection fraction
Source: Eur J Heart Fail. 2025 Sep 16;27(12):2800–9. doi: 10.1002/ejhf.70047 (PMC12803581; doi:10.1002/ejhf.70047)
Supplement: Supplementary file 1 — Appendix S1. Supporting Information. [file EJHF-27-2800-s001.docx]

**Supplemental materials**

***Supplemental Table 1: Association of ITE with outcome overall and by treatment group in Cox survival models***

|  | ***Cohort = RALES and EMPHASIS-HF*** | | ***Cohort = EPHESUS*** | |
| --- | --- | --- | --- | --- |
|  | ***HR (CI 95 %)*** | ***p-value*** | ***HR (CI 95 %)*** | ***p-value*** |
| *Effect of ITE (per 5 pts increase)* |  |  |  |  |
| *Overall* | *1.30 (1.21 - 1.40)* | *<0.0001* | *1.39 (1.30 - 1.48)* | *<0.0001* |
| *Placebo* | *1.40 (1.27 - 1.54)* | *<0.0001* | *1.51 (1.38 - 1.65)* | *<0.0001* |
| *MRA* | *1.16 (1.03 - 1.30)* | *0.011* | *1.26 (1.15 - 1.39)* | *<0.0001* |
|  | *Interaction p-value:* | *0.014* | *Interaction p-value:* | *0.007* |

***Supplemental Table 2: Interaction analysis of ITE and ITE components with MRA.***

| ***ITE component*** | ***Analysis in RALES and EMPHASIS-HF*** | | ***Analysis in EPHESUS*** | |
| --- | --- | --- | --- | --- |
|  | ***P-value for interaction*** | | ***P-value for interaction*** | |
|  | ***ITE x MRA***  ***in model 1**** | ***variable x MRA***  ***in model 2***** | ***ITE x MRA***  ***in model 1**** | ***variable x MRA***  ***in model 2***** |
| *Age* | *0.013* | *0.42* | *0.007* | *0.001* |
| *Weight* | *0.006* | *0.002* | *0.011* | *0.051* |
| *SBP* | *0.013* | *0.034* | *0.008* | *0.44* |
| *Heart rate* | *0.014* | *0.80* | *0.007* | *0.38* |
| *LVEF* | *0.013* | *0.44* | *0.019* | *0.29* |
| *eGFR* | *0.009* | *0.39* | *0.009* | *0.11* |
| *Potasium* | *0.014* | *0.35* | *0.007* | *0.49* |
| *Sodium* | *0.007* | *0.69* | *0.006* | *0.11* |
| *Diabetes* | *0.012* | *0.27* | *0.006* | *0.57* |
| *Stroke* | *0.014* | *0.40* | *0.008* | *0.66* |
| *Hypertension* | *0.020* | *0.56* | *0.007* | *0.31* |
| *All components* | *0.005* | *-* | *0.006* | *-* |

**Model 1 included ITE, MRA, interaction between ITE and MRA, and ITE component listed in the first column (or all variables for last line).*

***Model 2 included variable, MRA and interaction between ITE component listed in the first column and MRA.*

***Supplemental Table 3: Absolute treatment effect at 2 years according to ITE tertiles in the derivation and validation cohort***

|  | **Risk at 2 years (CI 95%)** | | | |
| --- | --- | --- | --- | --- |
|  | **Placebo** | **MRA** | **Risk difference**  **(CI 95%)** |  |
| Derivation cohort **(RALES + EMPHASIS-HF)** |  |  |  |  |
| Tertile 1: <= 8.9 | 0.354 (0.313 to 0.395) | 0.271 (0.233 to 0.310) | -0.083 (-0.139 to -0.026) |  |
| Tertile 2: ]8.9 ; 12.1] | 0.380 (0.339 to 0.422) | 0.312 (0.272 to 0.351) | -0.069 (-0.126 to -0.011) |  |
| Tertile 3 : > 12.1 | 0.500 (0.459 to 0.542) | 0.326 (0.286 to 0.367) | -0.174 (-0.232 to -0.116) |  |
| Validation cohort **(EPHESUS)** |  |  |  |  |
| Tertile 1: <= 6.7 | 0.190 (0.158 to 0.222) | 0.179 (0.154 to 0.204) | -0.011 (-0.052 to 0.030) |  |
| Tertile 2: ]6.7 ; 10.2] | 0.279 (0.237 to 0.320) | 0.234 (0.202 to 0.265) | -0.045 (-0.097 to 0.007) |  |
| Tertile 3 : > 10.2 | 0.351 (0.314 to 0.387) | 0.286 (0.250 to 0.323) | -0.064 (-0.115 to -0.013) |  |

***Supplemental Table 4. Individual Treatment Effect Calculator for MRA Therapy***

***Supplemental Figure 1: Association of ITE according to treatment group in derivation and validation cohorts.***

*
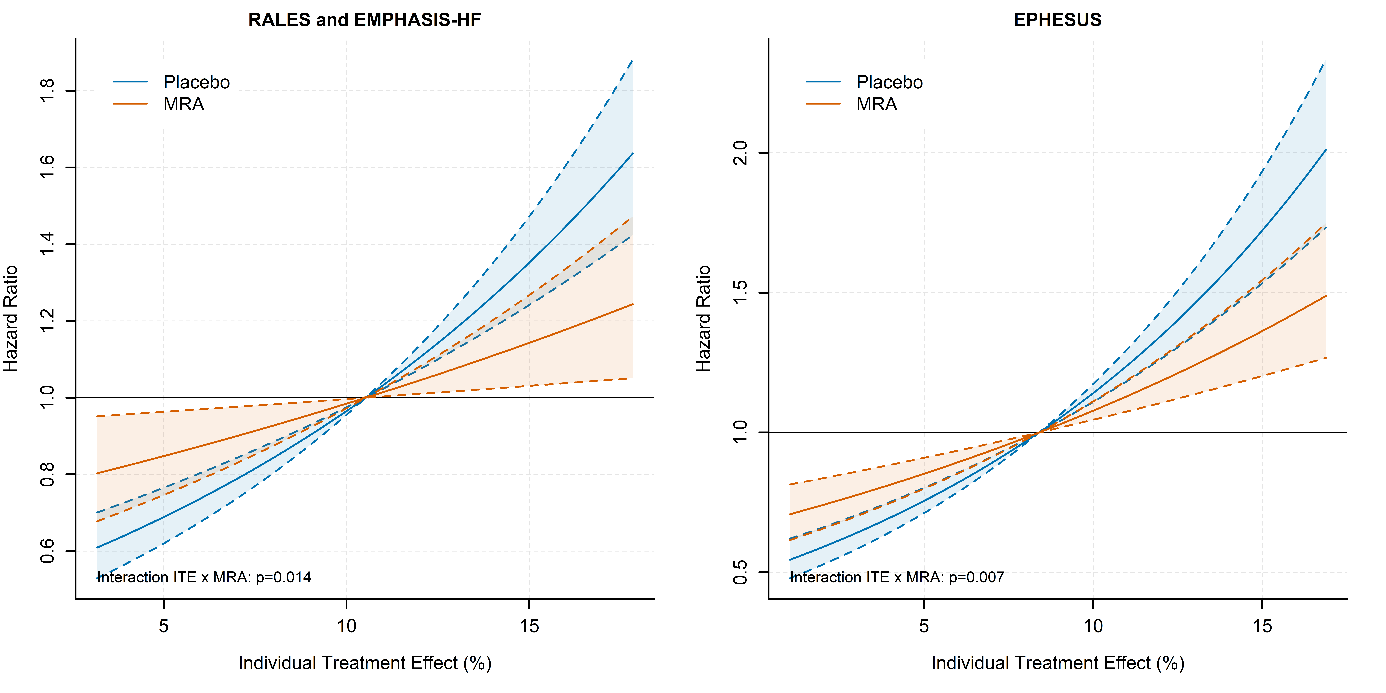
*
